# A Panel of Potential Serum Markers Related to Angiogenesis, Antioxidant Defense and Hypoxia for Differentiating Cutaneous Squamous Cell Carcinomas from Actinic Keratoses

**DOI:** 10.3390/jpm14010103

**Published:** 2024-01-17

**Authors:** Simona Roxana Georgescu, Sandra Milena Tocut, Clara Matei, Corina Daniela Ene, Ilinca Nicolae, Mircea Tampa

**Affiliations:** 1 Department of Dermatology, ‘Carol Davila’ University of Medicine and Pharmacy, 020021 Bucharest, Romania; simona.georgescu@umfcd.ro (S.R.G.); mircea.tampa@umfcd.ro (M.T.); 2 Department of Dermatology, ‘Victor Babes’ Clinical Hospital for Infectious Diseases, 030303 Bucharest, Romania; drnicolaei@yahoo.ro; 3 Faculty of Medicine, Tel-Aviv University, Tel Aviv 69978, Israel; milena.tocut@wmc.gov.il; 4 Department of Internal Medicine, “Wolfson Medical Center”, 61 Halochamim Street, 58100 Holon, Israel; 5 Department of Nephrology, ‘Carol Davila’ Nephrology Hospital, 010731 Bucharest, Romania; 6 Departments of Nephrology, ‘Carol Davila’ University of Medicine and Pharmacy, 020021 Bucharest, Romania

**Keywords:** cSCC, AK, markers, angiogenesis, oxidative stress, hypoxia

## Abstract

Cutaneous squamous cell carcinoma (cSCC) arising from the malignant proliferation of epidermal keratinocytes is the second most common skin cancer. Actinic keratosis (AK), which is considered cSCC in situ, may progress into invasive tumors. Currently, there are no serum markers that can differentiate cSCC from AK. The aim of our study was to assess angiogenesis and oxidative stress in patients with cSCC and patients with AK and find reliable serum markers useful in the diagnosis of cSCC. We have determined the serum levels of a group of proangiogenic factors (MMP-2, MMP-9, VEGF, FGF2), the total antioxidative status/capacity (TAS/TAC), ImAnOx, a marker of oxidative stress, and HIF-1 alpha, an indicator of hypoxia. We have identified higher serum levels of MMP-2. MMP-9, VEGF, FGF2 and HIF-1 alpha and lower levels of ImAnOx in cSCC patients compared to AK patients and controls. There were no statistically significant differences between AK patients and controls. We have found positive correlations between proangiogenic markers and HIF-1 alpha and negative correlations between proangiogenic markers and ImAnOx. Our results suggest that MMP-2, MMP-9, VEGF, FGF2, ImAnOx and HIF-1 may be promising markers for differentiating AK from cSCC, and there is a link between angiogenesis, oxidative stress and hypoxia.

## 1. Introduction

Cutaneous squamous cell carcinoma (cSCC) represents the second most common type of skin cancer, surpassed only by basal cell carcinoma (BCC) [1]. cSCC is an important public health issue, imposing a significant burden on healthcare systems worldwide. Its incidence is continuously increasing due to the progressive aging of the global population [2]. cSCC can develop from actinic keratoses (AK), encountered in 14% of the population [3], with incidence increasing with the age of the individual. These premalignant lesions are mostly due to the life-long exposure of skin keratinocytes to UV radiation and are considered in situ squamous cell carcinoma. However, not all AKs transform into invasive cSCC, as the rate of frank malignant transformation ranges from 0.025% to 16% [4,5]; due to the large number of AK cases in the general population, this results in an astonishing number of 1.8 million new cases of cSCC reported annually [6].

The overall aggressiveness across cSCC patients may seem relatively reduced compared to other cancers, as the cSCC metastasis rate varies between 2 and 9.9% in the immunocompetent population, with a 2.8% chance of dying because of this disease; when considering the large number of individuals affected, one can easily observe that the number of deaths caused by cSCC is very high [7,8]. Thus, the number of deaths caused by cSCC rivals the number of deaths from melanoma [6]. When caught early, most SCCs are curable, hence the need for a tool to predict the malignant transformation of AK into cSCC. Not all patients are amenable to biopsy/surgical procedures, as cSCC usually occurs in aged patients, some of whom have various comorbidities that contraindicate surgery and preclude excision interventions. Not all AKs transform into cSCC, and since multiple AKs are usually present in a large number of lesions for each patient, surgical excision and evaluation for all lesions is costly and not feasible [9], hence the imperative need to have serum markers that can differentiate between AK and cSCC.

Angiogenesis is a complex process that involves the formation of new vessels. It is a process that takes place in several stages, including the stimulation of endothelial cells by growth factors, their proliferation, the release of angiogenic factors from the extracellular matrix through the action of degradative enzymes and the development of the first vessels (capillary formation). This process is governed by numerous proangiogenic mediators such as vascular endothelial growth factor (VEGF), fibroblast growth factor 2 (FGF2), metalloproteinases (MMPs), etc., and antiangiogenic factors (endostatin, thrombospondin, angiostatin, etc.) released by endothelial cells, immune cells and tumor stroma cells. It is widely recognized today that angiogenesis is an essential process in tumor growth, with the new blood vessels playing a pivotal role in supplying nutrients necessary for tumor proliferation [10,11]. The interplay between cancerous cells and newly developed blood vessels represents a crucial biochemical pathway for the expansion of solid tumors and the formation of metastases. Understanding the angiogenesis pathways involved in carcinogenesis may represent the basis for new targeted therapies [12].

There is a close relationship between oxidative stress and tumor angiogenesis. Reactive oxygen species (ROS) induce the expression of VEGF in various cells including endothelial cells, smooth muscle cells and macrophages, which promotes angiogenesis. VEGF in turn enhances the synthesis of ROS in endothelial cells by activating NADPH oxidases. Lipid oxidation products have been shown to promote angiogenesis through the hypoxia-inducible factor (HIF) signaling pathway. There are also VEGF-independent mechanisms by which ROS stimulate angiogenesis, for example through the ROS/ATM/p38-alpha pathway [13]. The aim of our study was to assess angiogenesis and oxidative stress in patients with cSCC and patients with AK and find reliable serum markers useful in the diagnosis of cSCC. To achieve these goals, we have analyzed the serum levels of a group of proangiogenic factors (MMP-2. MMP-9, VEGF, FGF2), ImAnOx, a marker of oxidative stress, and HIF-1 alpha, an indicator of hypoxia. In addition, we have investigated the link between angiogenesis and oxidative stress and hypoxia.

## 2. Materials and Methods

### 2.1. Study Participants

We performed a case–control study over a period of three years and included consecutive patients with cSCC (n = 47), consecutive patients with AK (n = 47) and 40 age- and sex-matched controls (healthy subjects). To form the control group, we enrolled otherwise healthy subjects who attended the clinic of dermatology for dermatological conditions (such as skin tags or nevi) that do not influence the levels of the studied markers. All study participants signed an informed consent form, and the study respects the ethical standards in the Declaration of Helsinki. The study protocol was approved by the Ethics Committee of the Victor Babes Infectious and Tropical Diseases Hospital, Bucharest, Romania (13050/31.07.2017). To confirm the diagnosis, we performed histopathological examinations for each patient with AKs or cSCC.

The inclusion criteria were subjects over 18 years, without systemic diseases, with a satisfactory nutritional status, with no treatment for cSCC or AKs. The exclusion criteria consisted of conditions that can interfere with the levels of the markers we have determined: tobacco use, drug abuse, alcoholism, pregnant women, subjects under treatment with immunosuppressants, anti-inflammatory drugs, vitamins/nutritional supplements.

Blood samples were obtained following a 12 h fasting period using a holder–vacutainer system. Samples that were hemolyzed or appeared milky were not accepted. Subsequently, the samples were centrifuged at 3000× *g* for 10 min. The samples were stored at −80 °C.

### 2.2. Histopathological Examination

**Figure 1:**
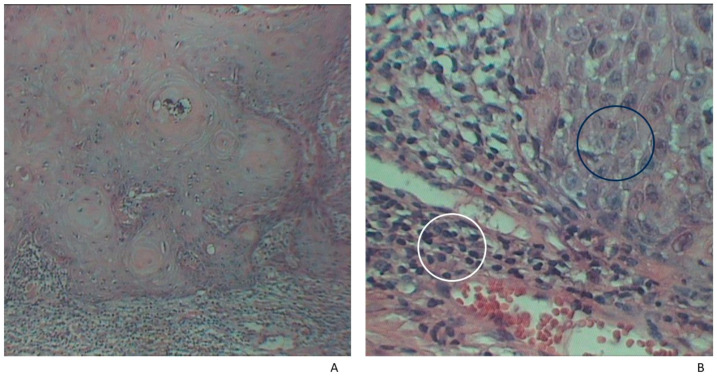
Histological examination of cSCC. (**A**) Aberrant proliferation of squamous cells exhibiting giant nuclei and nuclear polymorphism (black circle) accompanied by an abundant inflammatory infiltrate (white circle). (**A**) HE × 40, (**B**) HE × 90.

**Figure 2:**
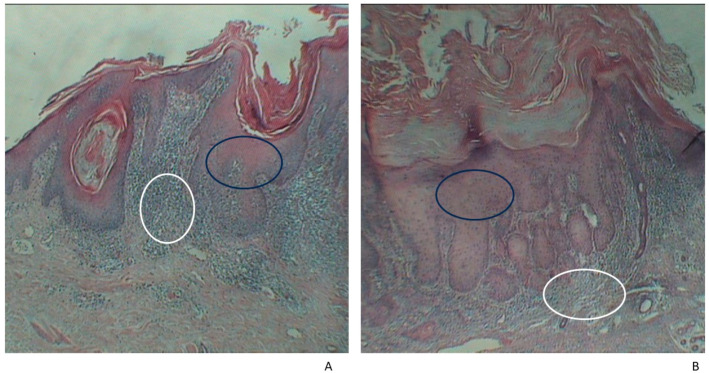
Histopathological examination of AK. Abnormal growth, with the presence of dysplastic keratinocytes (black circle) and inflammatory infiltrate (white circle) (**A**,**B**) HE × 40.

The tissue samples were preserved in 10% formalin and then processed using the paraffin-embedding technique. A hematoxylin–eosin stain (HE) was used for microscopic analysis (Figure 1 and Figure 2). The volume of the sample collected for the histological analysis varied according to tumor size (0.4–2.5 cm).

### 2.3. Serum Marker Determinations

Enzyme-linked immunosorbent assay (ELISA) was employed to determine the serum concentrations of MMP-2, MMP-9, VEGF, FGF2 and HIF-1 alpha, and for ImAnOx, a photometric assay was used. The utilized ELISA kits were specifically designed for research purposes, and we performed the tests according to the manufacturer’s protocol.

MMP-2 levels (ng/mL) were determined using the sandwich ELISA method (ELISA kit ELH-MMP-2, RayBiotech, Peachtree Corners, GA, USA) with a good sensitivity (3.5 pg/mL). The results were expressed as ng/mL and read using a colorimetric TECAN analyzer (wavelength 450 nm).

MMP-9 levels (ng/mL) were determined using the sandwich ELISA method (ELISA kit ELH-MMP-2, RayBiotech, Peachtree Corners, GA, USA) with a good sensitivity (10 pg/mL). The results were expressed as ng/mL and read using a colorimetric TECAN analyzer (wavelength 450 nm).

VEGF levels (pg/mL) were determined using the sandwich ELISA method (ELISA kit DVE00 RD Systems, Minneapolis, MN, USA), a sensible technique—sensitivity 9 pg/mL. The colorimetric assessment of the final product was performed at a wavelength of 450 nm, using a colorimetric reader (semi-automatic TECAN analyzer).

FGF2 (pg/mL) was measured using the sandwich ELISA method (ELISA kit DFB50, RD Systems, Minneapolis, MN, USA), a sensible technique—sensitivity 3 pg/mL. The colorimetric assessment of the final product was performed at a wavelength of 450 nm, using a colorimetric reader (semi-automatic TECAN analyzer, Mannedorf, Switzerland).

ImAnOx (µmol/L) evaluates the total antioxidative status/capacity (TAS/TAC) in serum. The assessment of the antioxidative capacity was made through the reaction between the antioxidants in the sample and the hydrogen peroxide that we added. The limit of detection was 130 µmol. We used a photometric assay.

HIF-1alpha (ng/mL) was determined using the sandwich ELISA method (MyBioSource, San Diego, CA, USA). The limit of detection was 0.01 ng/mL. The colorimetric assessment of the final product was performed at a wavelength of 450 nm, using a colorimetric reader (semi-automatic TECAN analyze, Mannedorf, Switzerland r).

### 2.4. Statistical Analysis

For the statistical analysis, we used the Kruskal–Wallis test and the Dunn post hoc test for a triple comparison of the groups. The data distribution was assessed using a Kolmogorov–Smirnov test. The correlation between parameters was performed using Spearman’s correlation coefficient. The significance level (*p*) was established at 0.05 (5%) and the confidence interval at 95% for hypothesis testing.

## 3. Results

Table 1 presents the characteristics of study participants and Table 2 includes the features of cSCCs and AKs diagnosed in the patient groups.

The serum levels of the proangiogenic markers (MMP-2, MMP-9, VEGF, FGF2) and HIF-1 alpha (a marker of hypoxia) were higher in cSCC patients compared to AK patients and controls. The serum levels of ImAnOx, a marker of oxidative stress, were lower in cSCC patients compared to AK patients and controls. There were no significant differences in the AK group compared to the control group (Table 3).

There were negative correlations between proangiogenic parameters (MMP-2, MMP-9, VEGF, FGF2) and ImAnOx. Positive correlations were identified between proangiogenic factors and HIF-1 alpha (Table 4).

There were no statistically significant correlations between the tumor characteristics (diameter, depth of invasion and ulceration) and the studied parameters.

## 4. Discussion

Finding reliable markers for the diagnosis of cSCC would obviate the need for an excisional biopsy of every suspicious lesion, which is currently hardly feasible or acceptable and costly. Up until now, there have been no serum markers that can differentiate between AK and cSCC.

MMPs are zinc-dependent endopeptidases that are involved in numerous processes such as embryonic development, tissue remodeling, angiogenesis and carcinogenesis. Currently, there is widespread recognition that MMPs exert their action on a diverse array of substances, encompassing membrane receptors, cytokines, growth factors, signaling molecules, etc. MMPs are seen more as regulators of cellular signaling rather than solely as degradative enzymes. Functioning as angiomodulators, MMPs participate in activating proangiogenic factors and contribute to the development of new blood vessels—an essential support system for tumor growth and progression. Consequently, recent studies emphasize MMPs’ role in fundamental stages of carcinogenesis, including tumor progression, angiogenesis, invasion and metastasis, suggesting their potential as target molecules for preventing and treating neoplasms [14,15,16,17,18].

MMP-2 is a gelatinase secreted mainly by fibroblasts, myofibroblasts and cardiomyocytes. It is a versatile molecule that acts on a large plethora of substrates such as collagen, versican elastin, fibronectin endothelin, osteopontin, MMP-9, MMP-13, plasminogen, etc. [14,19]. It has been shown to be highly expressed in the initial stages of cSCC [20]. Fundyler et al. were the first to demonstrate a correlation between increased levels of MMP-2 and inflammation, angiogenesis and tumor invasion in cSCC [21]. Regarding the link between MMP-2 and tumor grade, the results are conflicting [22,23]. De Oliveira Poswar et al. highlighted that MMP-2 expression is higher in cSCC compared to BCC, and the findings were similar when the authors compared cSCC with AK. MMP-2 expression correlated with the tumor grade in BCC and AK but not in cSCC [22]. However, Hernandez Perez et al. found a correlation between cSCC grade and MMP-2. These conflicting results could arise from variations in tissue fixation methods and antigen retrieval techniques. Additionally, the differences in race and genetics within distinct populations might impact the expression of MMP-2 across various grades of SCC [24]. In the current study, we identified higher serum levels of MMP-2 in cSCC patients compared to AK patients and controls. The differences were not significant when we compared the AK patients with the control group. A recent study showed that after the topical treatment of AK with different treatments such as 0.8% piroxicam cream associated with sunscreen, photodynamic therapy or ingenol mebutate gel, there was a significant reduction in the expression of metalloproteinases (MMP-1 and MMP-2) in the lesional tissue [25].

In our study, the results obtained for MMP-9 were similar to those obtained in the case of MMP-2. MMP-9 is a gelatinase that cleaves basement membrane components such as type IV collagen, a major component of it. The enzyme is secreted especially by inflammatory cells in the tumor microenvironment (neutrophils, mast cells, macrophages) and less by tumor cells [20]. Haji et al. compared the expression of the metalloproteinases MMP-7, MMP-8 and MMP-9 in oral SCC and cSCC, in tumoral and peritumoral tissue. They observed increased expression of MMP-7 in oral SCC compared to cSCC, which was expected considering that MMP-7 is particularly expressed in mucosa. MMP-8 and MMP-9 were highly expressed in peritumoral inflammatory tissue [26]. These findings are consistent with those obtained by Pettersen et al., who highlighted on cSCC samples that tumor-associated macrophages (TAMs) abundantly expressing CD163 release significant amounts of MMP-9 [27]. A study comparing cSCC and keratoacanthoma samples revealed the presence of CD163+ macrophages and MMP-9+ cells only in cSCC samples [28]. Thus, it can be stated that the main source of MMP-9 in the tumor microenvironment is represented by TAMs. Furthermore, TAMs release COX-2, which in turn stimulates the release of VEGF and FGF [20]. The expression of MMP-9 by immunosuppressive TAMs is associated with tumor aggressiveness [28].

Poswar et al. analyzed the immunohistochemical expression of MMP-9 in BCC, cSCC and AK and identified the highest levels in cSCC. No correlation was observed between MMP-9 expression and Broders’ or Bryne’s grading systems [29]. Although Lee et al. claim that MMP-9 is a more sensitive marker than MMP-2 for the diagnosis of cSCC, performing studies on both human and murine tissues, additional studies are needed to obtain conclusive data [30]. Previous studies determining the serum levels of MMP-9 in patients with head and neck SCC (HNSCC) have shown that these individuals present higher levels compared to the control group, both preoperatively and postoperatively, without a correlation with the histological type or clinical characteristics of the tumor [31,32]. Additionally, another study indicated that preoperative serum MMP-9/TIMP-1 and MMP-2/TIMP-2 ratios were higher in patients with HNSCC compared to the control group [33]. The results of our study showed higher serum levels of MMP-2 and MMP-9 in cSCC patients compared to those with AK and the control group. The two enzymes exert a degradative effect on the ECM and have proangiogenic activity; therefore, they contribute to the more aggressive behavior of cSCC compared to AK. These data also explain why there were no statistically significant differences between AK patients and the control group, AK representing cSCC in situ. These results taken together suggest that increased expression of MMP-2 and MMP-9 represents an early event in cSCC carcinogenesis.

Angiogenesis is a key factor in the process of tumor growth, local invasion and metastasis. VEGF family members (VEGF-A, VEGF-B, VEGF-C, VEGF-D, VEGF-E and placental growth factor (PlGF)) are among the most important proangiogenic factors, involved in the proliferation of new vessels from existing ones. VEGF stimulates the proliferation of endothelial cells with the formation of new vessels, promoting vascular permeability and inflammation [34]. VEGF is a main actor in tumor angiogenesis, its level being elevated through the expression of oncogenes, various growth factors and hypoxia [35].

A recent review focused on the investigation of the factors involved in angiogenesis in cSCC and its precursors. Many of the studies indicated an increased expression of VEGF, especially in the advanced stages of the tumor [36]. Previous studies showed that VEGF expression correlated with the degree of tumor differentiation. Thus, VEGF expression is higher in moderately or poorly differentiated cSCCs compared to well-differentiated forms [37,38]. It is known that poorly differentiated tumors are associated with deeper tissue invasion, which attracts a higher risk of metastasis. That is why VEGF could represent a prognostic factor, associated with the risk of metastasis. Strieth et al. showed that the mean vascular density is similar in normal skin tissue and in AK, with only a slight increase being observed in the case of hypertrophic AKs and early-stage cSCC. Late-stage cSCC exhibited abundant vascularization [39].

The main mechanisms proposed for the increase in VEGF expression in HNSCC are the activation of HIF signaling due to hypoxia at the level of the tumor mass, genetic alterations and the activation of various signaling pathways by inflammatory cells and fibroblasts that are present in the tumor microenvironment [40]. The discovery that VEGF is also secreted by non-endothelial cells has provided new insights in the field of angiogenesis. It has been postulated that keratinocytes are the main VEGF-secreting cells in the skin and to a lesser extent macrophages and fibroblasts. VEGF can act directly on keratinocytes, influencing their proliferation and survival, thus participating directly in the process of skin carcinogenesis [41].

Studies investigating the levels of VEGF in the blood of patients are few. Srivastava et al. identified higher serum levels of VEGF-A in patients with HNSCC compared to the control group but also in patients with more advanced stages of the disease (stages III and IV) compared to patients with tumors in stages I and II [42]. Siemert et al. suggest that the level of VEGF in plasma can be considered a potential prognostic marker in patients with HNSCC [43]. In our study, we measured the serum levels of VEGF and obtained statistically significantly higher values in patients with cSCC compared to the control group but also compared to the group of AK patients. There were no significant differences between the control group and AK patients. The high levels identified in cSCC patients suggest that VEGF can be considered a factor that modulates tumor development, being a key molecule in the angiogenesis pathway.

FGF2 belongs to the FGF family of heparin-binding growth factors and is expressed in various tissues, exhibiting a broad range of biological activities. FGF2 orchestrates the physiological process of new vessel formation but also pathological angiogenesis. FGFs act as mitogenic factors by stimulating the proliferation of fibroblasts and endothelial and cancer cells. The FGF receptor family consists of four members (FGFR-1, FGFR-2, FGFR-3 and FGFR-4), which are mainly involved in collagen synthesis [44,45]. Dysregulation of FGFRs’ functions seems to be involved in numerous cancers; evidence indicates that FGF contributes to cSCC development [46].

Studies on endothelial cell cultures have shown that FGF2 acts as a proangiogenic factor by stimulating the proliferation and migration of endothelial cells and the expression of specific integrins [44]. Hertzler-Schaefer et al. suggested that autocrine activation of FGF signaling is a key event in SCC tumorigenesis [47]. Previous research has shown that FGF2 stimulates VEGF expression in endothelial cells [48]. The metabolic activity of neoplastic cells has a crucial role in the process of metastasis and angiogenesis. Neoplastic cells secrete large amounts of lactate, which is taken up by endothelial cells, an event that promotes FGF2 and VEGFR signaling, which contribute to angiogenesis [49]. In a controlled in vitro environment, Jee et al. showed that FGF and COX-2 act as downstream factors in the angiogenic activity induced by IL-6 in BCC cells. The increase in FGF2 expression due to IL-6 is facilitated by the activation of the JAK/STAT3 and PI3-Kinase/Akt pathways [50].

Recent data support that the FGFR axis influences the activity of keratinocytes, which may lead to the appearance of AK [51]. Increased FGF2 mRNA expression was identified in the dermal segment of AK [52]. In HNSCC, it has been shown that tumor cells, through the secretion of FGF2, promote the activation of the FGF2/FGFR2 axis in cancer-associated fibroblasts (CAFs) in the dermis. This, in turn, plays a role in activating CAFs and induces mTOR-mediated autophagy, a process associated with the release of pro-tumorigenic factors [53]. FGF2 and its receptors FGFR2 and FGFR3 have been proposed as markers of malignant transformation in precancerous oral lesions [45].

Data on serum FGF2 levels in cSCC are limited. In our study, we identified increased serum levels of FGF2 in cSCC patients compared with AK patients and controls, with no differences between AK and controls. Therefore, FGF2 could represent a promising marker and therapeutic target in the diagnosis and management of cSCC.

Various proangiogenic factors are associated with oxidative stress. Under physiological conditions, ROS are involved in the process of normal angiogenesis. Nevertheless, elevated concentrations of ROS could contribute to pathological angiogenesis. Angiogenesis induced by oxidative stress plays a significant role in the advancement of cancer and chronic diseases [13]. The involvement of oxidative stress in skin diseases is a hot topic, and recent research has provided valuable information in this field, opening new avenues toward efficient diagnostic methods and innovative, personalized therapies [54,55,56,57]. However, data on markers of oxidative stress in cSCC are limited. Studies have evaluated markers of oxidative stress in cSCC, using serum or tissue samples. Elevated levels of oxidants have been observed in the serum of patients compared to the control group [58,59]. Additionally, higher levels of prooxidant molecules and lower levels of antioxidants have been observed in tumor tissue compared to adjacent healthy tissue [60]. A study conducted in 2020 including patients with AK, Bowen disease and cSCC revealed that DNA oxidation contributes to the initial phase of carcinogenesis, while protein oxidation is implicated throughout all stages of carcinogenesis, and lipid oxidation becomes particularly relevant in the later stages of the process [61].

We determined ImAnOx, a marker of serum antioxidant capacity, and identified lower levels among cSCC patients compared to AK patients and controls. There were no statistically significant differences between AK patients and the control group. It has been shown that antioxidants can downregulate angiogenesis [62]. We have investigated the link between proangiogenic factors (MMP-2, MMP-9, VEGF and FGF2) and ImAnOx. We have identified statistically significant negative correlations between ImAnOx and all four proangiogenic factors studied, which demonstrates that the antioxidant defense in cSCC patients was exceeded.

One of the most important stimuli of angiogenesis is hypoxia. Under hypoxic conditions, tumor cells induce the expression of HIF-1 alpha, which activates VEGF, promoting angiogenesis. Additionally, HIF-1 alpha induces the expression of MMP-2 and MMP-9 [20]. HIF-1 alpha modulates angiogenesis by regulating the activity of VEGF-A and VEGF receptors (VEGFR1, 2 and 3), FGF2 and inducible nitric oxide synthase [63]. Considering the lack of vascularization in the epidermis, it can be considered that at this level, there is a slightly hypoxic microenvironment, therefore there are increased levels of HIF-1 alpha that mediate the response of keratinocytes to UVB. HIF-1 alpha activity is closely related to numerous processes such as cell proliferation, apoptosis, cell migration, repair processes and angiogenesis [64]. According to a study by An et al. the expression of HIF-1 alpha and VEGF is higher in cSCC compared to Bowen’s disease and seborrheic keratoses, and an association between HIF-1 alpha expression and advanced histological grade of cSCC was also observed [65]. Conversely, the expression of prolyl hydroxylase domain protein 2 (PHD2), an inhibitor of HIF-1 alpha, was increased in normal skin compared to lesional skin [65]. An et al. performed the first study to evaluate the PHD/HIF-1 alpha axis in the pathogenesis of SCC. This pathway is involved in tumor growth, the formation of new vessels and resistance to radiotherapy and chemotherapy [65]. Hypoxia induces proliferation and aberrant differentiation of keratinocytes. Inhibition of HIF-1 alpha is associated with a decreased ability of tumor formation and malignant transformation in keratinocytes [64,66]. The higher expression of HIF-1 alpha in cSCC compared to BCC could be due to the more aggressive behavior of cSCC, leading to a hypoxic environment, which is an important stimulus for the expression of HIF-1 alpha [67]. Under low-oxygen conditions, several factors including ROS can stabilize HIF-1 alpha, which supports cell invasion and evasion of the immune system and resistance to radiation, which denotes the link between hypoxia and oxidative stress [68].

In the current study, we identified higher serum levels of HIF-1 alpha in cSCC patients compared to AK patients and controls. The differences were not significant when we compared AK patients with the control group. HIF-1 alpha might be involved in the pathogenesis of cSCC by regulating the adaptation of tumor cells to conditions of hypoxia generated by increased cell proliferation. We have also analyzed the relationship between proangiogenic factors (MMP-2, MMP-9, VEGF and FGF2) and HIF-1 alpha and observed statistically significant positive correlations between HIF-1 alpha and all four angiogenic factors, which supports the link between angiogenesis and hypoxia. Therefore, HIF-1 inhibitors may represent a promising adjuvant therapy to enhance the effectiveness of existing cancer therapies.

## 5. Conclusions

The results of the present study indicate higher serum levels of the proangiogenic factors MMP-2, MMP-9, VEGF and FGF2 and HIF-1 alpha, an indicator of hypoxia, and lower serum levels of ImAnOx, a marker of oxidative stress, in cSCC patients compared to AK patients and the control group. In AK patients, no significant changes in these markers were identified. The studied parameters may represent potential markers for the differentiation of cSCC from AK and decrease the number of histopathological examinations that are invasive, expensive and time-consuming. The present study indicates a direct link between angiogenesis and hypoxia on the one hand and angiogenesis and oxidative stress on the other hand, which may be the basis for the identification of new therapies.

## Figures and Tables

**Table 1:** Study participant characteristics.

Group Characteristics	cSCC Patients (47 Cases)	AK Patients (47 Cases)	Control Group (40 Patients)	*p* Value
Age (years)	58.1 ± 11.4	56.7 ± 15.2	55.2 ± 12.5	*p*> 0.05
Female/male	29/18	27/20	23/17	*p* > 0.05
Body mass index (kg/m^2^)	24.2 ± 2.7	23.5 ± 3.2	23.8 ± 4.2	*p* > 0.05
Glycemia (mg/dL)	91.5 ± 12.3	94.5 ± 9.5	90.7 ± 8.3	*p* > 0.05
Hemoglobin (g/dL)	13.4 ± 1.1	13.1 ± 0.9	12.4 ± 0.7	*p* > 0.05
Creatinine (mg/dL)	0.8 ± 0.3	0.9 ± 0.2	0.8 ± 0.2	*p* > 0.05
Urea (mg/dL)	23.3 ± 6.6	22.7 ± 7.5	21.5 ± 9.3	*p* > 0.05
C-reactive protein (mg/dL)	4.2 ± 1.1	0.6 ± 0.3	0.5 ± 0.2	*p* < 0.05

cSCC—cutaneous squamous cell carcinoma; AK—actinic keratosis. The results are expressed as mean and standard deviation.

**Table 2:** Histological characteristics of AK and cSCC samples.

Tumor Characteristics	cSCC (47 Cases)	AK (47 Cases)
Diameter <2/>2 cm	20/27	47/0
Depth of invasion <4/>4 mm	21/26	47/0
Ulceration (present/absent)	36/11	1/46
Disease history (years)	2.2 ± 1.7 *	5.1 ± 4.0 *

cSCC—cutaneous squamous cell carcinoma; AK—actinic keratosis. * The results are expressed as mean and standard deviation.

**Table 3:** The serum levels of the studied markers expressed as mean and standard deviation.

Parameter	cSCC Group	AK Group	Control Group	*p* *	*p* **
MMP-2 (ng/mL)	1157.55 ± 221.65	449.25 ± 52.01	452.87 ± 88.62	<0.01	A vs. B: <0.01 A vs. C: <0.01 B vs. C: 0.71
MMP-9 (ng/mL)	519.40 ± 140.77	172.19 ± 14.24	168.67 ± 27.83	<0.01	A vs. B: <0.01 A vs. C: <0.01 B vs. C: 0.72
VEGF (pg/mL)	473.19 ± 95.96	119.51 ± 18.86	126.1 ± 29.23	<0.01	A vs. B: <0.01 A vs. C: <0.01 B vs. C: 0.23
FGF-2 (pg/mL)	42.89 ± 11.07	7.63 ± 1.13	6.77 ± 2.59	<0.01	A vs. B: <0.01 A vs. C: <0.01 B vs. C: 0.16
ImAnOx (μmol/L)	252.40 ± 122.17	294.80 ± 36.17	302.17 ± 12.01	<0.01	A vs. B: <0.01 A vs. C: <0.01 B vs. C: 0.013
HIF1-alpha (ng/mL)	134.25 ± 27.19	68.59 ± 6.87	58.7 ± 18.65	<0.01	A vs. B: <0.01 A vs. C: <0.01 B vs. C: 0.075

cSCC—cutaneous squamous cell carcinoma; AK—actinic keratosis; MMP-2—metalloproteinase 2; MMP-9—metalloproteinase 9; VEGF—vascular endothelial growth factor; FGF2—fibroblast growth factor 2; ImAnOx—TAS/TAC, antioxidative capacity; HIF-1 alpha—hypoxia-inducible factor 1 alpha; *p* *—triple comparison of the groups; *p* **—pairwise comparison of the groups. The results are expressed as mean and standard deviation.

**Table 4:** Correlations between proangiogenic markers and ImAnOx and HIF-1 alpha in cSCC group.

Parameter	MMP-2		MMP-9		VEGF		FGF2	
	rho	*p*	rho	*p*	rho	*p*	rho	*p*
ImAnOx	−0.85	<0.01	−0.71	<0.01	−0.73	<0.01	−0.82	<0.01
HIF-1 alpha	0.72	<0.01	0.86	<0.01	0.77	<0.01	0.60	<0.01

MMP-2—metalloproteinase 2; MMP-9—metalloproteinase 9; VEGF—vascular endothelial growth factor; FGF2—fibroblast growth factor 2; ImAnOx—TAS/TAC, antioxidative capacity; HIF-1 alpha—hypoxia-inducible factor 1 alpha.

## Data Availability

All data are contained within the article.
